# Comparison between acute kidney injury (AKI) and non‐AKI patients secondary to severe hypothyroidism

**DOI:** 10.1002/ccr3.1393

**Published:** 2018-04-17

**Authors:** Bing Han, Tong Cheng, Lin Ye, Chunhua Sui, Lizhen Yang, Dongping Lin, Jie Qiao, Yingli Lu

**Affiliations:** ^1^ Institute and department of Endocrinology and Metabolism Shanghai Ninth People's Hospital Affiliated to Shanghai Jiaotong University School of Medicine Shanghai China

**Keywords:** Acute kidney injury, creatine kinase, globulin, hypothyroidism, rhabdomyolysis

## Abstract

Hypothyroidism was a rare cause of rhabdomyolysis, which finally progressed to acute kidney injury (AKI). We compared nine patients with AKI secondary to hypothyroidism and six patients with severe hypothyroidism. Besides creatine kinase, globulin could be an alternative biomarker of rhabdomyolysis related to hypothyroidism.

## Introduction

Hypothyroidism has become more common in the general population 1 and is characterized by elevated serum levels of thyroid‐stimulating hormone (TSH); hypothyroidism can be divided into two categories based on thyroxine (T4) or triiodothyronine (T3) levels: overt hypothyroidism and subclinical hypothyroidism. Hypothyroidism has been reported as a rare cause of rhabdomyolysis, which is caused by muscle necrosis and the leakage of myoglobin in muscle cells. It causes tubular obstruction and increased intraluminal pressure thus decreasing glomerular filtration. Moreover, free radicals are produced by heme‐induced oxidative damage to the renal tubule, which also promotes the development of acute kidney injury (AKI).

Acute kidney injury represents the clinical condition in which the renal excretory function is critically reduced, and the body is unable to maintain metabolic balance. It could be caused by various etiologies, including endocrine disorders. AKI caused by hypothyroidism is a rare complication and has not attracted enough attention; this complication is commonly associated with prolonged hospitalizations and long‐term morbidities 2.

Acute kidney injury secondary to hypothyroidism is induced by rhabdomyolysis. One of the main features of rhabdomyolysis is increased creatine kinase (CK) levels and serum thyroid hormone levels that are inversely related to CK levels in hypothyroid patients 3. Thus, elevated plasma CK levels were thought to be the most sensitive marker of muscle injury 4. However, there are no comparisons of CK with other serum biomarkers in hypothyroidism‐induced AKI.

In our study, we describe fifteen cases of severe hypothyroidism patients, including nine patients with hypothyroidism combined with AKI. Furthermore, we compared the differences between the two groups to identify other sensitive biomarkers of rhabdomyolysis related to hypothyroidism.

## Case Presentation

We retrospectively collected fifteen inpatients with severe hypothyroidism (TSH >50 IU/mL) from January 2009 to June 2014 in the Shanghai Ninth People's Hospital affiliated to Shanghai Jiaotong University School of Medicine. Among these patients, nine had AKI secondary to hypothyroidism. Blood samples were gathered from all patients after fasting for at least 8 h and were centrifuged at 2000 rpm for 15 min at room temperature. All biochemical indexes were analyzed using a BECKMAN COULTER AU 680 and an original kit. Thyroid function was measured using a BECKMAN COULTER DXI800. Hypothyroidism was diagnosed by symptoms and thyroid hormone. Serum creatinine levels greater than 115 μmol/L were defined as impaired kidney function.

The AKI group included four males and five females (age 59.78 ± 19.36 years), and the non‐AKI group included six females (age 56.67 ± 15.65 years). In the AKI group, two patients did not have edema, two patients had pericardial effusion, four patients had edema of the face and limbs, and one patient had limb edema. However, in the non‐AKI group, two patients were without edema, three had facial edema, and one patient had lower limb, eyelid, hand, and pleural effusion. One patient in the AKI group had hypertension (level 1), and two patients in the non‐AKI group had hypertension (level 1). However, there were no significant differences in the systolic (123.3 ± 14.1 mmHg vs. 126.7 ± 20.7 mmHg, *P* > 0.05) or diastolic (76.7 ± 10.3 ± 14.1 mmHg vs. 78.3 ± 7.5 mmHg *P* > 0.05) blood pressures between the AKI and non‐AKI groups, respectively. Besides that, there was no significant difference of urine specific gravity and urine PH between two groups.

In the AKI and non‐AKI groups, the creatinine levels were significantly different (141.67 ± 38.86 vs. 82.17 ± 13.24 μmol/L, *P* < 0.01). The CK, CKMB, and globulin levels were significantly different between these groups (*P *=* *0.0271, 0.0092, and 0.0177, respectively). However, there were no differences in the blood lipids, electrolytes, albumin, LDH, GPT, GOT, or bilirubin levels (Table 1).

**Table 1:** Comparison of biomarkers between hypothyroidism patients with and without acute kidney injury (AKI)

	AKI	Non‐AKI	*P* value
Creatinine, μmol/L	141.67 ± 38.86	82.17 ± 13.24	**0.0033**
Uric acid, μmol/L	374 ± 131.49	276.5 ± 45.90	0.1074
CHOL, mmol/L	7.32 ± 2.70	6.70 ± 1.64	0.8017
TG, mmol/L	2.07 ± 1.24	1.62 ± 0.64	0.4357
HDL, mmol/L	1.37 ± 0.39	1.51 ± 0.38	0.5375
LDL, mmol/L	4.66 ± 2.22	4.49 ± 1.20	0.8695
K, mmol/L	3.67 ± 0.37	3.92 ± 0.18	0.1481
Na, mmol/L	139.33 ± 4.42	139.17 ± 5.31	0.9482
Cl, mmol/L	98.89 ± 3.69	99.67 ± 3.61	0.6934
CO_2_CP, mmol/L	27.4 ± 3.22	23.43 ± 1.61	**0.0159**
LDH, U/L	364.89 ± 226.79	187 ± 39.28	0.1562
CK, U/L	1371.33 ± 949.57	130.75 ± 74.15	**0.0271**
CKMB, U/L	37.22 ± 21.15	3 ± 2.16	**0.0092**
Albumin, g/L	42.33 ± 5.05	38.67 ± 5.92	0.2202
Globulin, g/L	30 ± 3.84	37.67 ± 7.15	**0.0177**
r‐GT, U/L	37.33 ± 26.87	54 ± 47.48	0.3984
GPT, U/L	43.44 ± 30.89	47.67 ± 17.67	0.7679
GOT, U/L	59.44 ± 32.06	32.67 ± 8.80	0.0699
AKT, U/L	61.33 ± 13.12	67.67 ± 25.12	0.5311
Total bilirubin, μmol/L	12.33 ± 0.04	10.17 ± 0.41	0.6070
Conjugated bilirubin, μmol/L	3.11 ± 2.09	2 ± 0	0.2205
Bile acid, μmol/L	9.01 ± 3.20	6.62 ± 2.27	0.1386

Bold values represent significant difference between two groups.

**Figure 1:**
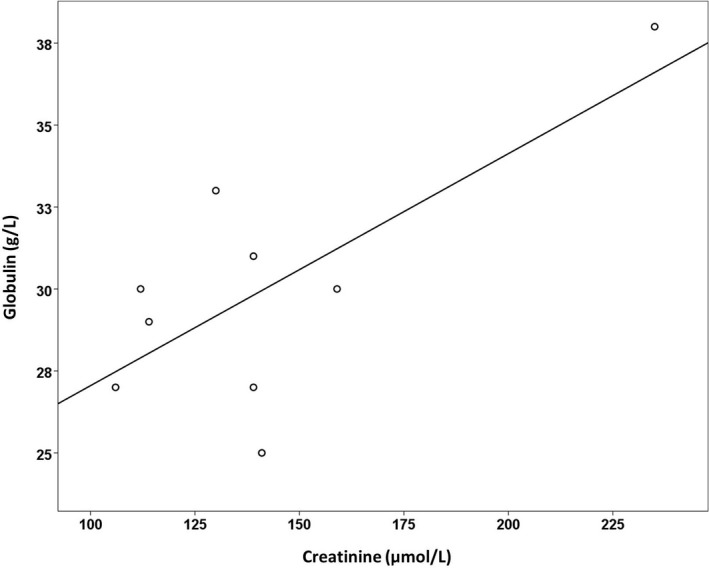
Pearson correlation of globulin with creatinine. The result indicated creatinine levels were positively correlated with globulin levels (*r* ^2^ **=* *0.716, *P *=* *0.03).

For the AKI group, Pearson's correlation was used to analyze the relationship between the creatinine level and the other indexes, such as the CK, CKMB, and globulin levels. The results indicated that creatinine levels were positively correlated with globulin levels (*r* ^2^ **=* *0.716, *P *=* *0.03) (Fig. 1).

## Discussion

In the present study, we compared nine hypothyroidism patients with AKI and six without AKI. The CK, CKMB, and globulin levels significantly differed between the two groups. Furthermore, Pearson's correlation indicated that the creatinine levels were positively correlated with the globulin levels. In the AKI group, all patients had elevated CK levels, but none had muscle pain.

Many conditions could cause rhabdomyolysis, including traumatic muscular injury and various metabolic, connective tissue, rheumatologic, and endocrine disorders 5. In our patients, AKI was due to rhabdomyolysis caused by severe hypothyroidism.

In addition to hypothyroidism, other endocrine disorders such as hyperthyroidism, diabetic ketoacidosis (DKA), hyperosmolar hyperglycemic state (HHS), hyperaldosteronism, and even pheochromocytoma have been reported to cause rhabdomyolysis 4, 5.

Rhabdomyolysis‐induced AKI is a rare complication of hypothyroidism. Most of the literature presents case reports or small sample summaries. The clinical manifestation of rhabdomyolysis varies from elevated CK levels to severe conditions such as electrolyte imbalances and acute renal failure. Cai et al. 6 reported five cases of AKI secondary to hypothyroidism‐induced rhabdomyolysis. All patients had the condition combined with facial and lower limb edema. Ahmed et al. 7 even reported a patient with Hoffman's syndrome (proximal muscle weakness and pseudohypertrophy) caused by hypothyroidism, which was accompanied by rhabdomyolysis and AKI.

Rhabdomyolysis caused by hypothyroidism is unusual. It is proposed that thyroid hormone deficiency‐related glycogenolysis and mitochondrial oxidative metabolism are possible reasons underlying rhabdomyolysis 8. Serum CK is the gold standard for diagnosing rhabdomyolysis 6. For a long time, CK levels were considered to be predictive of developing AKI. CK levels of more than 5000 U/L have been related to renal failure 9, but not renal failure severity 10. In our study, the CK, CKMB, and globulin levels were significantly different between hypothyroidism patients with and without AKI. However, Pearson's correlation indicated that the globulin levels were significantly correlated with the creatinine.

Acute kidney injury is a critical syndrome with high cost and is defined as a rise in creatinine levels of ≥0.3 mg/dL (26.4 μmol/L) or ≥50% from the baseline value within 48 h, or a decrease in urine output below 0.5 mL/kg/h for 6 h or more 11. This is known as AKIN criteria established in 2007. Chertow et al. 12 indicated that a rise in creatinine levels of more than 0.3 mg/dL was independently associated with an approximately fourfold increase in hospital mortality in 9210 inpatients.

As the incidence of hypothyroidism increases, AKI induced by hypothyroidism deserves more attention. Early diagnosis and treatment can improve the prognosis and prevent late‐stage complications in these patients. Thyroxine replacement therapy is the primary treatment, and the prognosis is good. Regular treatment includes dieresis, alkalinization of the urine, and stopping the use of drugs such as amiodarone 6. Some patients even require renal replacement therapy 13.

In our study, all the patients were given a levothyroxine replacement. In patients with AKI, we used fluid infusion and alkalizing agents. Holt et al. suggested early aggressive fluid replacement with saline is beneficial 14. Vikrant et al. reported renal impairment secondary to hypothyroidism, which is reversed after thyroid hormone replacement therapy 15.

There are also some limitations. This is a retrospective analysis. Some patients cannot be followed up. Hypothyroidism may occur in different stages. The peak CK value is observed at 12–24 h after the onset of rhabdomyolysis. Because the admission times of the patients varied, the peak value of CK may have passed. Besides, we also lack long‐term follow‐up.

## Conclusion

Rhabdomyolysis‐induced renal injury was a severe complication of hypothyroidism. We reported the largest population of AKI due to hypothyroidism caused by rhabdomyolysis. Globulin levels were positively correlated with creatinine levels and could be used as an indicator of renal failure.

## Consent

Written informed consent was obtained from the patient for publication of this case report.

## Conflict of Interests

None declared.

## Authorship

YL and JQ: designed and supervised this investigation. BH and TC: performed this investigation. LY and CS contributed to the data collection. LY and DL: provided technical or material support. All authors read and approved the final manuscript.
